# In Silico Forensic Toxicology: Is It Feasible?

**DOI:** 10.3390/toxics13090790

**Published:** 2025-09-17

**Authors:** Ivan Šoša

**Affiliations:** Department of Anatomy, Faculty of Medicine, University of Rijeka, 51 000 Rijeka, Croatia; ivan.sosa@uniri.hr

**Keywords:** artificial intelligence, break-even analysis, forensic toxicology, in silico forensic toxicology, integration

## Abstract

In silico forensic toxicology refers to the emerging application of computational models based on Quantitative Structure–Activity Relationships (QSARs), molecular docking, and predictions regarding Absorption, Distribution, Metabolism, Excretion, and Toxicity (ADMET) as used to predict the toxicological behavior of various substances, particularly in medico-legal contexts. These computational models replicate metabolic pathways, providing insights into the metabolism of substances in the human body, while the results of this approach effectively reflect the necessary compounds, reducing the need for direct laboratory work. This review aims to evaluate whether forensic settings and in silico methods present a cost-effective strategy for investigating unknown substances, aiding in toxicological interpretations, and steering laboratory process analyses. Additionally, financial considerations, such as break-even analysis and Bland–Altman plots, were conducted, indicating that forensic labs conducting over 625 analyses each year can achieve cost efficiency by integrating in silico strategies, thus making them a viable alternative to conventional methods in high-throughput settings. Recent studies have emphasized how machine learning enhances predictive accuracy, thereby boosting forensic toxicology’s capacity to effectively evaluate toxicity endpoints. In silico methods are essential for cases involving novel psychoactive substances (NPSs) or unclear toxicological findings. They are also useful as a supporting method in legal contexts, as they uphold expert testimonies and reinforce evidence claims. The future of forensic toxicology is likely to see the increased implementation of AI-powered techniques, streamlining toxicological investigations and enhancing overall accuracy in forensic evaluations.

## 1. Introduction

In silico toxicological experiments utilize computational models to simulate and predict the toxic effects of chemicals through digital representations of their molecular structures. Methods commonly used in drug discovery and risk assessment include techniques such as Quantitative Structure–Activity Relationships (QSARs) [1], molecular docking, and Absorption, Distribution, Metabolism, Excretion, and Toxicity (ADMET) estimates, which are principally used to rapidly assess potential adverse effects without the necessity for immediate laboratory testing.

The first mention of “in silico toxicology” was in the environmental chemistry and toxicology literature from 2010. Mekenyan’s anthological chapter “In silico toxicology: principles and applications”, in a book edited by Cronin and Madden [2], was the earliest documented use of the exact term in a book-length entry. A few years later, the term “in silico forensic toxicology” began to show up in the literature. Its earliest appearance, as identified by this literature review, is found in some conference papers from 2015 and 2016 [3,4]. Therefore, although this concept is relatively recent and has not been around for a long time, it is not entirely new.

In forensic toxicology, in silico techniques provide a quick and economical means with which to anticipate the effects of substances related to cases like poisoning and the detection of new psychoactive drug compounds [5]. They aid in interpreting data from complex biological matrices and in encountering substances with little or no historical toxicological data. In this realm, in silico tools help bridge that gap. Moreover, they have the capacity to simulate metabolic pathways in order to predict how a substance may transform within the human body. This is a critical factor when determining the cause of death or exposure during toxicological investigations. By predicting potential metabolites and interactions of substances of medico-legal interest, a forensic expert can more accurately interpret analytical results and determine the focus of subsequent in vitro or in vivo analyses. It is precisely this predictive capability that enhances its value in assessing whether the compound in question is rare or emerging, as traditional toxicological data might be nonexistent (as in cases of new psychoactive substances (NPSs)) [6,7].

**Figure 1:**
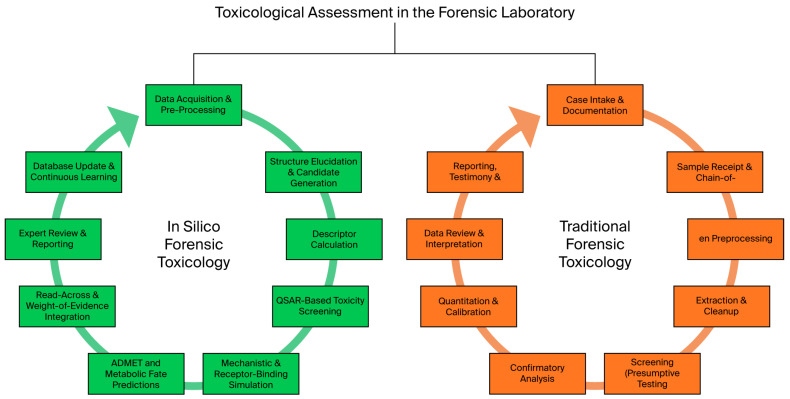
A typical workflow for applying silico methods to forensic toxicology.

A typical workflow for the use of silico methods in forensic toxicology is shown in Figure 1, beginning with thorough data curation, which involves collecting details about the chemical’s structure, related analogs, and any known toxicological endpoints [8,9].

Data curation is followed by typical in silico methods, model selection, and descriptor computation, where properties such as lipophilicity, electronic distribution, and steric factors are calculated. Afterwards, prediction algorithms such as QSAR models are used to predict toxicity endpoints, including acute toxicity, organ toxicity, and carcinogenicity. After completing in silico experiments, expert review and validation of the predictions are crucial to ensure that the computational outputs align with biological plausibility and real-world observations [10,11]. Ultimately, in silico forensic toxicology incurs unique expenses related to regulations and validation, which can offset potential cost savings [12,13]. However, it is crucial to understand that forensic evidence must conform to certain strict legal standards. In many EU jurisdictions, in silico results can currently be used only as a screening tool or as a supplement to traditional methods [14,15,16]. Complete regulatory validation must be obtained for full acceptance [17,18]. The costs associated with thorough validation can add to the initial investment; however, once the processes are established, significant savings can be observed [19,20].

Forensic toxicologists can integrate the results of in silico techniques and traditional analytical methods to build a more comprehensive picture of chemical hazards. For instance, when dealing with unknown samples from a postmortem analysis, computational predictions can guide the laboratory’s analytical focus, pointing out which metabolites to trace or which toxicological pathways to scrutinize.

In addition to enhancing the interpretation of toxicological profiles, in silico approaches also strengthen evidentiary bases in legal and regulatory contexts [20]. Additionally, structured approaches, such as those typically used in forensic toxicology laboratories, not only reduce the costs associated with experimental testing but also facilitate alignment with the 3Rs (replacement, reduction, and refinement) of animal use in toxicology [21]. As the field continues to evolve, the integration of machine learning with traditional computational models is likely to refine predictive accuracy further [22].

As cases of forensic toxicology become increasingly complex, particularly those involving novel substances with limited historical data, the need for predictive, computational approaches has become more critical than ever. While traditional toxicological analyses are reliable, they often demand substantial resources and time to yield conclusive results. On the contrary, in silico methods offer rapid assistance in forensic investigations.

This review aims to evaluate whether in silico methods can effectively be incorporated into standard forensic toxicology workflows, ensuring that they are both technically dependable and cost-effective. It involves a systematic literature review, assessment of model performance (Bland–Altman plot, ratios of contribution margins, and break-even analysis), and legal and regulatory acceptance.

## 2. Systematic Literature Review

**Figure 2:**
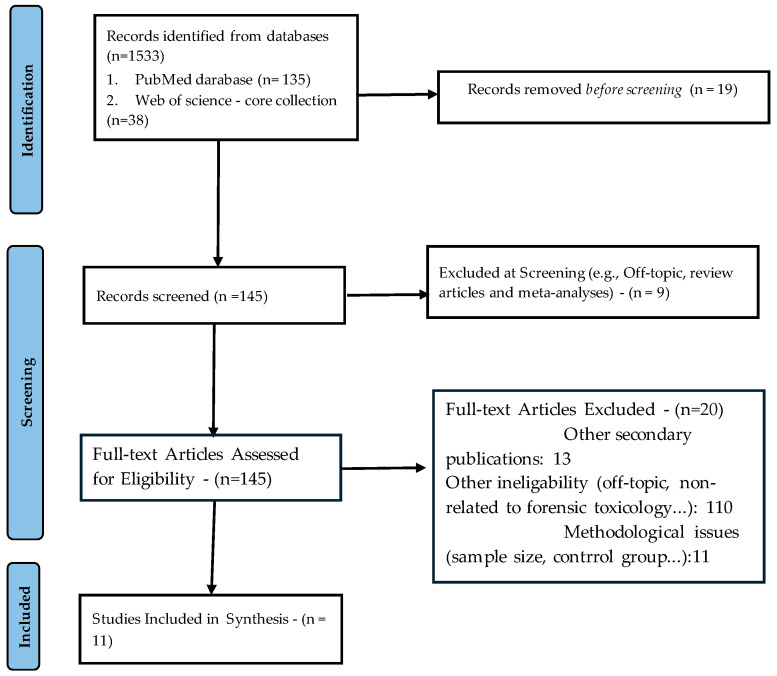
PRISMA 2020 flow diagram.

For this article, a literature search was conducted on two major databases—PubMed and Web of Science—from their inception to 3 June 2025. The search included all records.eligible for the search terms “in silico toxicology” AND “forensic” in any field. The search is outlined in the PRISMA 2020 flow diagram presented in Figure 2 (only relevant, methodologically sound primary studies on in silico forensic toxicology were included).

Independent queries in PubMed and Web of Science resulted in 173 records (search was conducted based on the all search fields). Removing 19 duplicates that could have altered the count improved the accuracy of the results (this was based on the author list/title/abstract). The titles and abstracts of each record were screened. Nine records were promptly excluded as they undoubtedly fell under the categories of review or meta-analysis articles. During the screening phase, 13 secondary publications, such as book chapters and position papers, were also excluded. In this phase, an additional 11 papers were excluded due to various methodological flaws (a heavy reliance on in silico predictions without adequate experimental validation, small study sample, etc.). Furthermore, 110 papers were found to be irrelevant or out of the scope of this paper. In summary, after examining the complete texts of 145 records, a thorough analysis (search for original research articles and case reports presenting novel in silico toxicology methods applied to forensic contexts, and employing QSAR, molecular docking, ADMET predictions, machine learning classifiers, or hybrid in silico/in vitro/in vivo workflows) led to the exclusion of additional 20 articles. Ultimately, 11 primary studies were included in the synthesis.

The Open Science Framework (OSF) designated 10.17605/OSF.IO/8E2N as the digital object identifier (DOI) for this review protocol.

### 2.1. Primary Studies

This literature review focused on PubMed and Web of Science searches for primary studies related to in silico forensic toxicology. Recent research topics include synthetic opioids (Wohlfarth et al. [23], Berardinelli et al. [24]) and organophosphates (Noga et al. [25], Pampalakis et al. [26]). At the same time, new psychoactive substances are usually presented as case reports (K. Jurowski and L. Niznik (2024) [27]), demonstrating how these tools are increasingly part of forensic workflows. By analyzing their contributions, challenges, and practical use, we can determine if in silico methods are genuinely suitable for daily forensic work. Jurowski and Krosniak (2024) [28] and Noga et al. (2024, 2025) [13,25] demonstrate how QSAR and acute toxicity models provide toxicity estimates in hours rather than weeks, thereby guiding emergency and threat assessments. Pelletier et al. (2023, 2025) [29,30], Busardò et al. (2022) [31], and Tang et al. (2025) [32] demonstrate that in silico platforms can accurately forecast major phase I/II metabolites, focusing expensive in vitro assays and in vivo confirmations on the most likely targets. Through minimizing animal use and reducing reliance on high-throughput lab screening, computational predictions prioritize the most hazardous compounds for detailed follow-up.

Multiple studies integrate in silico, in vitro, and in vivo data. For instance, Wohlfarth et al. [1] validated in silico-predicted metabolites against human microsomes and volunteer samples for the synthetic analgesic compound AH-7921. Pelletier et al. paired docking studies with hepatocyte assays for 4-Chloro-α-pyrrolidinovalerophenone (4-Cl-PVP) [29], while Busardò et al. combined models with clinical sampling to map acetazolamide elimination [31]. These hybrid workflows guarantee that computational predictions are not isolated; instead, they are hypotheses that are enhanced by empirical data, thereby increasing confidence in forensic conclusions.

Many QSAR tools struggle with novel scaffolds and unusual ring conformations (e.g., bicyclic organophosphates), meaning that designer opioids may fall outside of the training sets, thus yielding uncertain predictions. Computational findings must meet stringent validation standards. Without fully peer-reviewed protocols, expert testimonies risk being challenged as “junk science.” In silico methods may miss minor or unexpected metabolites. Overreliance without in vitro confirmation can lead to undetected biomarkers in casework. Table 1 provides a summary of the primary studies retrieved.

### 2.2. Trails in Forensic Medicine Operated by In Silico Forensic Toxicology

Clinical trials specifically designed to assess medico-legal cases using in silico forensic toxicology are relatively uncommon [19]. While research in in silico forensic toxicology progresses, the increased integration of computational tools into traditional lab methods is inevitable [34], requiring additional validation and a more seamless translation of complex simulation outcomes into legally relevant and straightforward expert opinions [11,35,36].

In Table 2, key characteristics of controlled or observational studies identified in the literature search are summarized. These studies demonstrate how in silico methods can enhance traditional laboratory work and provide additional insights in legal contexts.

With these methods, forensic laboratories can complement conventional techniques, improving the consistency and transparency of toxicological interpretations in medico-legal cases.

### 2.3. Case Studies Where In Silico Predictions Have Directly Influenced Forensic Conclusions

There are a couple of examples where in silico predictions have been applied in forensic contexts, highlighting an apparent case involving genetic analysis in sudden cardiac death (SCD). Recently, Alape-Ariza et al. (2025) presented a study that stands out as a prime example of such an approach [40]. In many forensic autopsies, especially those involving SCD, traditional pathology can fail to uncover apparent anatomical abnormalities. This frequently occurs with channelopathies and cardiomyopathies, conditions in which minimal or no structural changes are evident, yet in which a genetic predisposition can lead to fatal arrhythmia. Investigators performed next-generation sequencing (NGS) to identify variants in genes associated with cardiac function. Bioinformatic tools and algorithms—such as those predicting pathogenicity (using, for example, MutPred2 and protein–protein interaction analyses)—were then employed to evaluate whether these genetic variants could have a functional impact on cardiac ion channels or other critical proteins.

Worth et al. (2011) [41] provided “A Framework for assessing in silico Toxicity Predictions: Case Studies with Selected Pesticides”, with a primary regulatory focus. In that study, computational models were developed to predict chemical toxicity, which can inform forensic toxicology investigations in poisoning and exposure cases (primarily via a regulatory focus). The framework explained how QSAR models can predict toxicological effects for chosen compounds, such as pesticides. Likewise, there are cases of suspected chemical poisoning, where traditional toxicology may be limited by the complexities of chemical metabolism or low-level exposures and where in silico predictions provided insights into possible metabolic activation pathways [39,42,43]. This strengthens the argument for using in silico methods to predict potential toxicity outcomes. These predictions served as supplementary evidence when the laboratory data on actual tissue concentrations were confusing [44,45]. By comparing the computational profiles with the observed adverse effects, forensic investigators could better argue causation in medico-legal contexts. In essence, the model offered a mechanistic explanation that, when cross-validated with available laboratory data, helped strengthen regulatory and legal assessments of chemical exposures. The presented case studies illustrate how QSAR models can be used to predict the toxicological profiles of chemicals.

#### Forensic Impact

By demonstrating that specific variants were likely pathogenic, Alape-Ariza et al. (2025) [40] could support the hypothesis that a genetically driven cardiac event was the cause of death. This computational evidence directly influenced the forensic conclusion by providing a post-mortem molecular explanation, which was limited by the macroscopic evidence. Worth et al. (2011) further argued that, in poisoning cases or scenarios involving chemical exposure, such predictions can help forensic toxicologists determine the potential impact of a chemical when experimental testing is impractical or too time-consuming [41].

### 2.4. Case Reports in In Silico Forensic Toxicology

Although case reports of in silico forensic toxicology may not be as numerous as conventional forensic case studies, the literature includes examples where computational approaches have played a significant role in case interpretations and risk assessments. Additionally, various open access publications have featured articles that integrate these computational methods with case data. Some articles describe scenarios like drug-facilitated crimes or accidental overdoses where in silico predictions complemented traditional toxicological findings to offer a more comprehensive understanding of the substance’s effects [23,46,47]. These cases emphasize the direct benefits of in silico forensic applications, laying the ground for incorporating these techniques into standard forensic procedures.

Although conventional case reports often continue to rely on empirical data, the integration and validation of in silico methods are gaining traction, especially when used as a complementary tool [48,49]. This growing body of work suggests that, as computational models become more refined and validated against empirical data, we can expect more forensic case reports to include an in silico component in the interpretation of toxicological findings.

For instance, recent studies have used in silico methods to predict the toxicity profiles of NPSs. One notable example is the assessment of 4-chloromethcathinone (4-CMC), where researchers predicted acute toxicity (LD_50_), genotoxicity, cardiotoxicity, and the potential for endocrine disruption. They utilized computational models that provided critical insights into the substance’s forensic relevance and guided further empirical research [27].

In addition to NPS evaluations, in silico approaches have been utilized in the forensic assessment of opioid analogs [7]. Research on a new synthetic opioid, AP-238, demonstrated that various toxicity endpoints—such as organ-specific effects and interactions with the human ether-a-go-go-related gene (hERG) channel—could be predicted using a range of in silico methods [28]. These predictions not only aided in risk assessment but also helped forensic toxicologists to propose potential mechanisms of toxicity relevant to forensic investigations. There are also emerging reviews and conference proceedings that discuss the potential, limitations, and future directions of in silico forensic toxicology in real case scenarios. These discussions often highlight the efficiency of in silico methods in narrowing down suspects, predicting postmortem redistribution phenomena [50], and even in applying forensic entomotoxicology [51]. In this context, researchers model the exposure of insects to toxins to determine the time since death more accurately. The cost-effectiveness of the in silico approach in forensic toxicology has been confirmed [13].

The in silico approach in forensic toxicology is gaining attention due to its cost-effectiveness and efficiency. Traditional toxicology methods often depend on animal testing or laboratory experiments, which can be expensive, time-consuming, and ethically problematic. In contrast, in silico methods utilize computational models and databases to predict toxicological effects, thereby significantly reducing costs and accelerating analysis [49].

One of the main benefits of in silico toxicology is that it removes the need for animal testing, a concern that is becoming increasingly important due to ethical reasons [52,53]. Besides preventing animal testing, the in silico method reduces the need for costly lab equipment, leading to lower costs, another equally important benefit [54]. In addition, computational models efficiently analyze large datasets, reducing the time required for the in silico method. Advanced algorithms improve predictive accuracy and reliability [55,56].

### 2.5. Pricing

Pricing for in silico forensic toxicology can vary, and exact costs are not always publicly available for all institutions. The pricing is dependent on the complexity of the analysis, the software used, and the specific toxicological endpoints being evaluated. The cost comparison between traditional and in silico forensic toxicology depends on these same factors. Comparing traditional and in silico forensic toxicology in a university hospital laboratory in the EU, which processes fewer than 400 analyses per year, reveals variation by country, institution, and test complexity. However, some rough estimates outline the main cost drivers as well as a typical forecast.

Considerable costs are earmarked for the initial investments of both approaches [49,57]. While traditional forensic toxicology requires advanced, expensive instruments such as gas or liquid chromatography coupled with mass spectrometry (GC–MS, LC–MS/MS), in silico methods rely on computational models, simulation software, and databases (sometimes referred to as “non-testing” [58]). Although this is demanding in itself, there is an open investment for software development or licensing (which can range from a few thousand to tens of thousands of euros per year) [59]. On the other hand, traditional forensic toxicology requires routine maintenance for laboratory equipment and its calibration [60]. In addition, equipment depreciates over time, which influences the cost per analysis.

Analysis in traditional forensic toxicology requires chemicals, solvents, reagents, and consumables such as vials and filters. These costs can add several dozen euros per test, often adding even more when tests involve complex matrices or lower detection limits. Costs like those are negligible in in silico forensic toxicology. Since the process is computer-based, there is no need for chemical reagents or physical consumables. This absence of recurring material costs can significantly reduce the per-analysis expense.

In addition, laboratory staff dedicate a substantial amount of time to the pre-analytical or post-analytical phase of sample handling. They prepare the sample, operate the instrument, and take part in interpreting the data. In facilities with low throughput, analysis costs rise because skilled personnel are needed and heavy workloads can impede achieving economies of scale [44]. Conversely, running a simulation or predictive model demands less labor per analysis. Once the system is established and validated, personnel costs primarily consist of routine maintenance, periodic model updates, and quality validation procedures [61].

**Figure 3:**
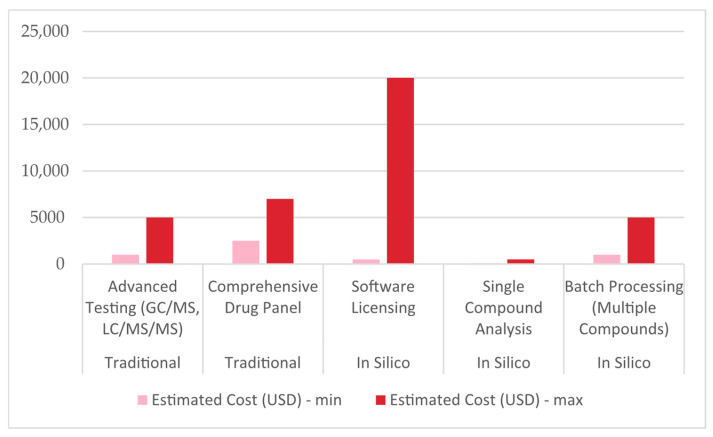
A chart comparing minimal and maximal prices (in USD) of traditional and in silico forensic toxicology. GC–MS, LC–MS/MS—gas or liquid chromatography coupled with mass spectrometry.

To reconcile the total estimated cost per analysis, equipment usage, consumables, and labor must be factored in, meaning the cost of traditional forensic toxicology analysis in a low-to-medium throughput setting in the EU may realistically be a few hundred euros. In the same setting, the cost of each in silico test might be a few dozen euros (approximately ten times less expensive, Figure 3) [62]. This makes in silico approaches desirable in environments where rapid screening or risk assessments are needed, provided that the legal and validation hurdles are appropriately managed.

This review considered a hypothetical model of 20 mid-throughput laboratories (each performing fewer than 1000 analyses annually), comparing their annual contribution margins as ratios of in silico to traditional forensic toxicology. The dataset was generated using Copilot, Version 1.0; Microsoft Corporation: Redmond, WA, USA, 2025; Available online: https://copilot.microsoft.com (accessed on 9 July 2025). The author has reviewed and edited, performing additional calculations by Microsoft Office 365 by Microsoft Corporation: Redmond, WA, USA, 2025; Available online: https://www.microsoft.com/en-us/microsoft-365 (accessed on 9 July 2025) All raw data, including the financial modeling spreadsheet, are provided in Datasheet S1 (Supplementary Materials).

The mean ratio when comparing traditional and in silico forensic toxicology, based on the annual ratios of contribution margins, was 0.996. This suggests that, on average, in silico services generate 99.6% of the revenue margin compared to traditional services across all 20 laboratories.

### 2.6. Break-Even Analysis

The fundamental break-even equation is as follows:

p×N=F+v×N

where

*p*: Revenue (or price charged) per analysis;
*N*: number of analyses;
*F*: Annual fixed costs (e.g., software licenses, infrastructure, maintenance);
*v*: Variable cost per analysis (e.g., additional materials, labor costs of analysts).

This calculates the number of analyses needed per year to make the in silico forensic toxicology approach profitable. Overall, it is crucial to assess yearly expenses in relation to the revenue generated from each analysis.

The total annual cost is

Total Cost=F+v×N.

The total annual revenue is

Total Revenue=p×N.

For the project to be profitable (or to, at least, break-even), the total revenue must equal or exceed the total costs.

p×N≥F+v×N.

A total number of analyses can be calculated using the following formula:

$$N=\frac{F}{p-v}$$

Assuming the relevant parameters for in silico forensic toxicology and traditional forensic toxicology are given in Table 3, then *F* = EUR 50,000 per year, *v* = EUR 20, and *p* = EUR 100 and, *F* = EUR 100,000 per year, *v* = EUR 80, and *p* = EUR 200, respectively [62,63,64].

**Figure 4:**
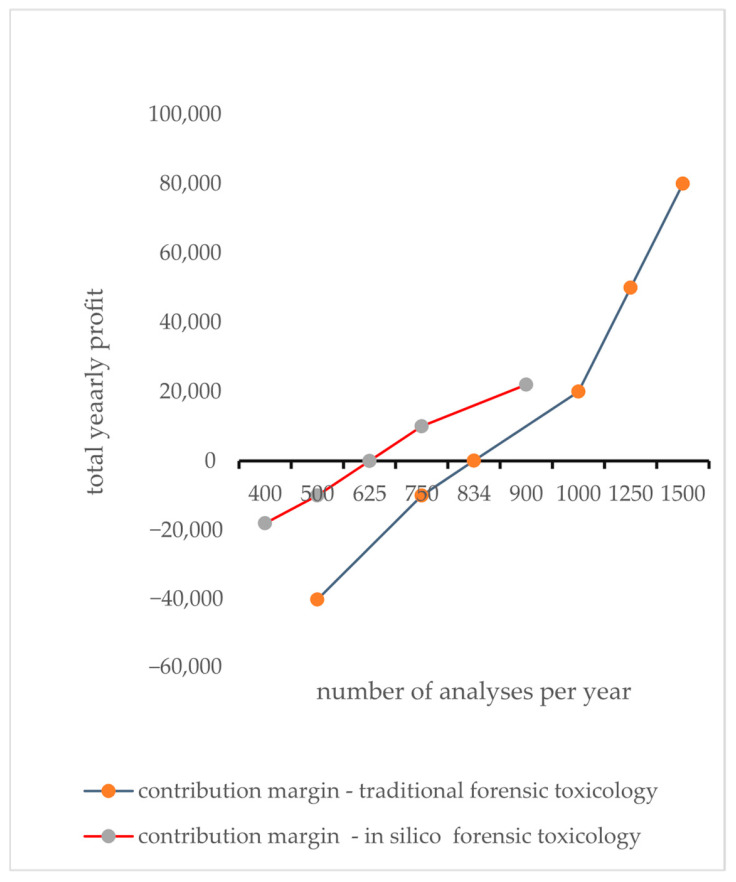
Break-even analysis serves as a strategic tool that identifies the exact point where fixed and variable costs intersect, resulting in profitability. It invites the audience to explore deeper financial insights that foster informed decision-making. The contribution margin, derived by subtracting variable costs from the selling price per unit, reveals how much revenue from each sale is available to cover fixed costs. A larger contribution margin indicates that each unit sold plays a greater role in achieving the break-even point. Once this threshold is achieved, every sale beyond this point adds directly to profit.

The in silico approach results in lower fixed costs (for software development, maintenance, and digital infrastructure) compared to physical lab equipment, as well as lower variable costs. This lower cost structure results in a lower break-even point (625 analyses) compared to the traditional method (Figure 4). While traditional methods may bring a higher per-analysis revenue—likely owing to the added value of established laboratory infrastructure and recognized accreditation—the contribution margin (revenue minus variable cost) is also higher (EUR 120 vs. EUR 80), which, in this sample, still requires a larger volume of tests (834 analyses) for the higher fixed costs to be covered.

### 2.7. Bland–Altman Plot

**Figure 5:**
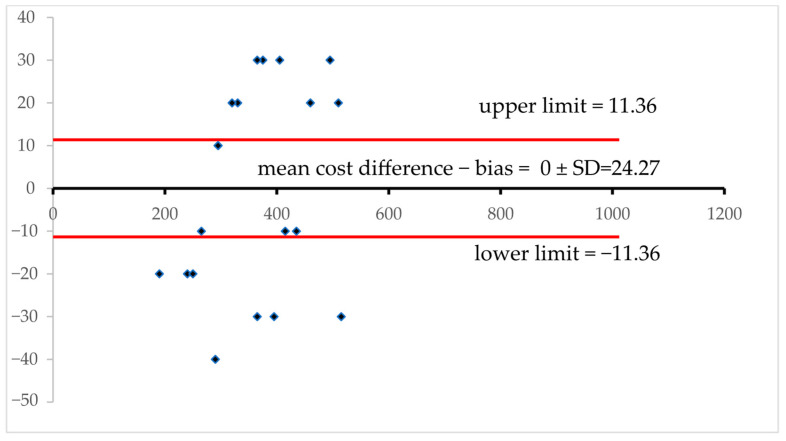
Bland–Altman plot assessing agreement between Method A and Method B across paired observations. Each data point represents the difference between the two methods plotted against their average value, allowing for the visualization of consistency and systematic bias. The central solid line represents the mean difference, commonly referred to as bias, which signals whether one method tends to yield higher or lower readings than the other. Flanking this, the upper and lower dashed lines demarcate the 95% limits of agreement—calculated as bias ±1.96 times the standard deviation of the differences—encompassing the range within which most discrepancies should fall if the methods are in reasonable concordance. This plot not only reveals the overall alignment between the two techniques but also exposes potential proportional bias if deviations vary across the measurement range. A uniform scatter around the bias line implies good agreement, while a widening spread or a visible slope suggests that error magnitude may change with increasing values—a phenomenon known as heteroscedasticity. Outliers beyond the dashed boundaries may pinpoint problematic measurements or method-specific weaknesses. Together, this visual framework provides an intuitive, data-rich assessment of whether Method A and Method B are interchangeable for analytical or clinical purposes.

A Bland–Altman plot was utilized to illustrate the cost comparison between traditional and in silico forensic toxicology, as presented in Figure 5. The dataset includes 20 cases of hypothetical laboratories processing a medium throughput of 1000 analyses per year, as well as at least that many forensic toxicology samples.

The mean cost difference, as represented by the central horizontal line, indicates the average difference between the two methods. The closer this value (line) is to zero, the more the methods align in cost. All data points fall between the upper and lower limits, represented by the dashed lines. Any values surpassing these lines indicate significant discrepancies. As there is no identifiable trend in the pattern of the assessed cost differences, which appear to be evenly distributed, these hypothetical cost differences are independent of the average cost, indicating a lack of systematic bias. Additionally, traditional and in silico methods show no comparable costs, as the data points do not cluster around the mean cost differences [65,66].

## 3. Future Insights

Both approaches complement and demonstrate a growing trend: in silico methods are used to fill the gaps left by traditional techniques. QSAR models for chemical exposures encompass a wide range of features. The rapid turnaround and scalability of these tools can be vital in medico-legal investigations. They facilitate fast and cost-effective toxicological profiling, as well as the prediction of genetic variants. These offer valuable insights in scenarios where conventional forensic medicine methods encounter obstacles [67,68]. Nonetheless, both approaches demand validation, meticulous documentation, and regulatory approval in order to be recognized as conclusive evidence (in the court) [20,69].

Genetic use has only been applied as an example; similar computational approaches are increasingly being explored in forensic toxicology, offering the potential to rapidly predict the biological impact of toxins without the need for extensive in vivo or in vitro testing. Since the current strategy involves integrating complementary features, the integration of in silico predictions in forensic investigations represents a broader shift. Specifically, there is a tendency to use computational biology to bridge the gaps left by conventional methodologies [70]. For instance, in cases of SCD, where the absence of clear anatomical pathology might otherwise leave questions unanswered, these methods provide a molecular-level insight that can be critical for legal determinations. As technology progresses, the forensic industry will increasingly incorporate integrated case studies where algorithms suggest causes of death and even boost experimental or circumstantial evidence in court [71].

### Machine Learning, Artificial Intelligence, and In Silico Forensic Toxicology

Recent acquisitions, including machine learning (ML) and artificial intelligence (AI), have profoundly transformed toxicology research [72]. Traditional forensic toxicology involved laboratories dedicating considerable time and resources to conducting in vivo or in vitro experiments aimed at investigating chemical hazards [73]. Today, in silico methods use computational power to simulate toxicological interactions, predict outcomes, and analyze vast datasets swiftly and reliably. This paradigm shift is especially significant in the setting where rapid, accurate assessments of toxins can have drastic consequences [19,74].

Forensic toxicology traditionally involves identifying and quantifying toxins in biological specimens. The aim is to establish the cause of death or behavior-related substance use. With the advent of ML and AI, researchers are now able to develop predictive models, such as QSAR models, to forecast the toxicity of chemicals without the need for extensive experimental testing. These in silico techniques can simulate how toxins interact with biological systems, offering insights into pharmacokinetic profiles via physiologically based pharmacokinetic (PBPK) models. It is even possible even to predict adverse outcomes. As noted in recent studies, machine learning is harnessed not only for classifying bioactivity (e.g., toxic vs. non-toxic) but also for exploring dose–response relationships [75], an approach particularly useful when dealing with the multifaceted data seen in forensic cases [76,77].

Modern AI methodologies, including deep neural networks, natural language processing, and ensemble learning techniques, excel in handling a wide array of datasets, from high-throughput screening results to intricate omics data [33]. In forensic toxicology, these methods effectively combine various data types (like spectral data, chemical structures, and clinical case histories) to anticipate toxic endpoints [28]. Although these tools offer significant benefits, their successful use depends on rigorous data quality management, clear model design, and ongoing collaboration among computational scientists and forensic experts.

Despite the promising advancements that ML and AI make within forensic toxicology, several challenges still persist. Therefore, it is crucial to validate various models and tailor them to specific forensic data [78,79]. Moreover, challenges such as the model’s accountability, biases in training datasets, and data sharing restrictions can delay regulatory approval and limit the broader implementation of forensic techniques [80]. As the domain progresses, there is an increasing need for user-friendly interfaces that allow both laboratory scientists and computational specialists to engage with these intricate systems, ensuring that technology enhances rather than complicates their tasks [81].

The combination of ML and AI extends well beyond forensic applications; for instance, these technologies are crucial in regulatory affairs toxicology [63,82], where they aid in chemical safety evaluations and risk management by providing quicker and more cost-effective toxicity assessments [1,83]. As the regulatory field transitions into an era driven by big data, insights derived from AI-enhanced models are expected to support more predictive, mechanistic-based approaches in toxicology [64]. Ultimately, these tools can simplify decision-making in public health, environmental protection, and chemical regulation, paving the way for innovations that transform preventive and investigative efforts in toxicology [84].

There is significantly more to explore, ranging from the development of data generation for rare compounds to emerging interdisciplinary collaborations [1,85,86,87]. Essentially, combining ML, AI, and in silico toxicology has a transformative impact on forensic and regulatory processes in toxicology [72]. As these areas converge, the faster detection and more advanced analysis of toxic substances can be expected. This will most likely be followed by the development of new benchmarks for scientific investigation and safety assurance [88]. Such a convergence is also prompting a broader discussion on the ethical, legal, and regulatory challenges of incorporating AI into traditionally empirical realms [89].

## 4. Limitations of In Silico Forensic Toxicology

Despite their growing importance, in silico methods in forensic toxicology have several critical limitations that must be recognized before their wide acceptance in routine use. To begin with, computational workflows may fail to predict minor or unexpected phase I/II transformation products [90]. Overreliance on these predictions without targeted in vitro confirmation may result in the omission of essential metabolites [91].

QSAR and molecular docking algorithms often struggle when confronted with novel scaffolds or unusual ring conformations—common features of designer drugs and bicyclic organophosphates [26]. Such compounds frequently lie outside the models’ training sets, resulting in low-confidence or conflicting toxicity estimates; the dependence of predictive accuracy on the representativeness and completeness of training datasets is also closely related to this issue. Proprietary algorithms and opaque data curation processes hinder independent review, while biases within the underlying data can systematically distort toxicity rankings.

Although in silico platforms eliminate consumable costs, they introduce significant fixed expenses. Licensing fees for specialized software, investment in server hardware, and ongoing model maintenance and retraining can all also be substantial. Moreover, skilled computational toxicologists are essential in managing, interpreting, and updating these complex systems. Economic analyses indicate that at least 625 annual analyses are needed to break-even; laboratories processing fewer than 400 cases per year cannot recover fixed costs, making traditional or immunoassay screening more cost-effective in low-volume settings.

Regulatory and legal challenges further constrain the routine use of in silico methods. In most EU jurisdictions, computational results are admissible only as screening tools or supplementary evidence. Full acceptance requires compliance with OECD QSAR validation principles, and the absence of standardized, peer-reviewed protocols renders expert testimony vulnerable to challenges in court [19,92].

Hybrid workflows—combining in silico, in vitro, and in vivo methods—offer the most scientifically robust approach but demand parallel experimental validation. The iterative cycle of predictive modeling, bench testing, and model refinement can erode anticipated time and cost savings, reducing the overall efficiency advantage of purely computational strategies.

Finally, restrictions on access to proprietary toxicological databases and the proliferation of “black-box” AI/ML models raise concerns about accountability, reproducibility, and privacy. These factors complicate both regulatory approval and defense of in silico findings under cross-examination.

Addressing these limitations through expanded chemical training sets, transparent validation frameworks, and harmonized reporting standards will be essential to advance in silico forensic toxicology toward routine, defensible use [93]. Ongoing dialog among computational scientists, laboratory toxicologists, and legal experts is crucial to overcoming both methodological and regulatory hurdles.

## 5. Conclusions

This review aims to evaluate whether integrating in silico methods into forensic toxicology workflows provides a cost-effective and reliable alternative to traditional analyses. We demonstrated that computational tools—such as QSAR, molecular docking, and ADMET predictions—can rapidly generate toxicity and metabolite profiles, guiding targeted in vitro and in vivo testing and reducing resource expenditure. Break-even analysis revealed that laboratories analyzing at least 625 samples annually can offset the fixed costs of software licenses, hardware, and model maintenance, achieving a per-analysis expense almost one-tenth that of chromatographic methods.

In high-throughput settings, in silico approaches excel at triaging NPSs and rare analytes, providing useful results within hours rather than weeks. They enhance legal evidence by contextualizing experimental data and predicting metabolites that might otherwise go undetected. However, full implementation relies on overcoming validation and regulatory barriers; models must satisfy OECD QSAR principles and adhere to standardized, peer-reviewed protocols. This is the major barrier to withstanding legal scrutiny. Overall, the hypothesis of the current paper is supported. When adequately empirically confirmed and used at a sufficient volume, in silico forensic toxicology is not only feasible but also economically valuable. Instead of replacing traditional toxicology methods, it acts as a valuable complement, streamlining workflows, saving resources, and improving the accuracy of medico-legal interpretations.

## Figures and Tables

**Table 1:** PICO table that summarizes the features of the included studies.

Study (Authors Year)	P: Population/Substance	I: Intervention/Methods	C: Comparison/Baseline	O: Outcomes	Reference
Wohlfarth et al., 2016	Human liver microsomes and reference volunteers exposed to AH-7921 In vitro metabolic stability assays; in silico prediction; in vivo confirmation	In silico predictions vs. observed in vitro/in vivo profiles	Metabolic stability data; full metabolite panel for AH-7921	Provides validated metabolites to target in forensic screens; improves interpretations of AH-7921 intoxications	[23]
Hernandez et al., 2019	Model chemical mixtures relevant to human exposures Integration of in vitro bioassays; in silico models; epidemiological data	Single-chemical risk assessments	Harmonized risk estimates for mixtures	Framework to interpret mixed-compound toxicology in forensic casework; supports expert testimony on combined exposures	[33]
Busardò et al., 2022	Human subjects and hepatic models given acetazolamide In silico metabolite prediction; in vitro hepatocyte assays; in vivo sampling	Conventional metabolic data for acetazolamide	Comprehensive metabolite list; elimination kinetics	Identifies masking agent metabolites for doping control; guides anti-doping laboratories’ workflows	[31]
Pelletier et al., 2023	Hepatic systems and volunteers exposed to 4-Cl-PVP Cross-disciplinary in vitro assays; in silico docking; limited in vivo profiling	Reference cathinone metabolic profiles	Structural identification of major and minor metabolites	Enables forensic labs to detect 4-Cl-PVP and differentiate it from other cathinones	[29]
Jurowski and Krosniak, 2024	New psychoactive substance AP-238 In silico QSAR and toxicity-prediction algorithms	Published toxicity endpoints of similar NPSs	Predicted LD50, ARfD, genotoxicity, organ toxicity endpoints	Rapid hazard-ranking tool for emergent NPSs; assists forensic toxicologists in triage and risk communication	[28]
Noga et al., 2024	Organophosphorus V-series nerve agents In silico acute-toxicity (LD_50_) modeling	Historical animal-derived LD_50_ values	Predicted human LD_50_ ranges	Supports threat assessments of chemical warfare agents; informs forensic readiness and triage protocols	[25]
Berardinelli et al., 2025	Novel synthetic opioid Dipyanone In vitro human hepatocyte incubations; in vivo volunteer studies; receptor binding assays	Methadone and known opioid metabolic profiles	Metabolite map; pharmacokinetic parameters; μ-opioid affinity	Supplies detection targets and potency data for forensic and clinical toxicology; refines interpretation of Dipyanone overdoses	[24]
Pampalakis et al., 2023,	Organophosphorus V-series nerve agents	In silico toxicity predictions (QSAR and computational models)	Empirical toxicity data from animal studies and the literature	Highlights critical limitations of unvalidated computational predictions in forensic assessments Warns clinicians of potential under-triage in V-agent exposures; emphasizes need for empirical confirmation	[26]
Noga and Jurowski, 2025	Bicyclic organophosphorus compounds In silico acute-toxicity and mechanistic models	V-series organophosphonates	Predicted LD50, mechanistic toxicity pathways	Guides forensic identification of emerging OP threats; supports rapid hazard evaluation	[13]
Pelletier et al., 2025	Diverse new psychoactive substances In silico metabolite prediction platforms	Experimental metabolite libraries	Ranked list of likely phase I/II metabolites	Prioritizes compounds for analytical method development in forensic labs; accelerates NPS detection	[30]
Tang et al., 2025	Two phenethylamine-derived NPSs Integrated in silico docking; in vitro microsomal assays; in vivo rodent studies	Standard phenethylamine metabolic pathways	Complete metabolite profiling; metabolic-kinetic parameters	Provides validated biomarkers for forensic screening of new phenethylamines; informs toxicological interpretation	[32]

QSAR—Quantitative Structure–Activity Relationship; L_50_—Median Lethal Dose (the dose that kills 50% of a test population, a benchmark for acute toxicity comparisons); ARfD—Acute Reference Dose.

**Table 2:** Characteristics of studies that involve controlled or observational processing employing in silico methodology.

Study	Design and Population	Intervention	Outcomes
Toennes et al. [37]	A controlled dosing study in human volunteers.	Oral administration of 4-fluoroamphetamine	Measurement of urinary metabolites (pharmacokinetics) to support forensic and therapeutic insights.
Papaseit et al. [38]	An observational study in humans evaluating acute effects.	Administration of mephedrone via oral and intranasal routes	Assessment of acute pharmacological effects (likely vital sign changes, subjective effects, etc.) in a real-life setting.
Losacker et al. [39]	A controlled, interventional pharmacokinetic study involving human subjects.	Controlled oral ingestion of 4-fluoroamphetamine	Measurement of chiral (R)/serum concentration (S) ratios to aid interpretation in forensic toxicology.

**Table 3:** Summary of fixed and variable cost assumptions for in silico and traditional forensic toxicology.

Parameter	In Silico Toxicology	Traditional Toxicology
Fixed Annual Costs (F)	EUR 50,000	EUR 100,000
Variable Cost per Analysis (v)	EUR 20	EUR 80
Revenue per Analysis (p)	EUR 100	EUR 200
Contribution margin per Analysis (p—−v)	EUR 80	EUR 120
Break-Even Analyses	625 analyses/year	834 analyses/year

## Data Availability

Available upon request from ivan.sosa@uniri.hr.

## Supplementary Materials

The following supporting information can be downloaded at: https://www.mdpi.com/article/10.3390/toxics13090790/s1, Datasheet S1 and Table S1.

## Abbreviations

The following abbreviations are used in this manuscript:

3Rs	Replacement, reduction, and refinement (of animal use)
4-Cl-PVP	4-Chloro-α-pyrrolidinovalerophenone (designer stimulant)
4CMC	4 Chloromethcathinone (a new psychoactive substance)
ADMET	Absorption, distribution, metabolism, excretion, and toxicity
AH-7921	Synthetic analgesic compound AH-7921
AI	Artificial intelligence
AP-238	An identifier for a new synthetic opioid discussed in the text
DOI	Digital object identifier
GC–MS	Gas chromatography–mass spectrometry
hERG	Human Ether-a-go-go-Related Gene
LC–MS/MS	Liquid chromatography–tandem mass spectrometry
LD_50_	Lethal dose for 50% of the test subjects
ML	Machine learning
NGS	Next-generation sequencing
NPS	Novel psychoactive substance
OSF	Open Science Framework
PBPK	Physiologically based pharmacokinetic (model)
PRISMA	Preferred reporting items for systematic reviews and meta-analyses
SCD	Sudden cardiac death
QSAR	Quantitative structure–activity relationship
