# Supplementary material for: In Silico Forensic Toxicology: Is It Feasible?
Source: Toxics. 2025 Sep 17;13(9):790. doi: 10.3390/toxics13090790 (PMC12474045; doi:10.3390/toxics13090790)
Supplement: Supplementary file 1 [file toxics-13-00790-s001.zip › Table S2.pdf]

Lab	Traditional_Contributor_Margin (kUSD)	InSilico_Contributor_Margin (kUSD)	Mean (kUSD)	Difference (kUSD)
Lab1	250	230	240	-20
Lab2	320	340	330	20
Lab3	410	380	395	-30
Lab4	290	300	295	10
Lab5	500	520	510	20
Lab6	380	350	365	-30
Lab7	450	470	460	20
Lab8	310	270	290	-40
Lab9	200	180	190	-20
Lab10	360	390	375	30
Lab11	420	410	415	-10
Lab12	260	240	250	-20
Lab13	480	510	495	30
Lab14	530	500	515	-30
Lab15	350	380	365	30
Lab16	270	260	265	-10
Lab17	390	420	405	30
Lab18	440	430	435	-10
Lab19	310	330	320	20
Lab20	290	300	295	10

Case	Traditional Cost (€)	In Silico Cost (€)	Mean Cost (€)	Difference (€)	
1	40000	800	20400	-39200	
2	35000	700	17850	-34300	
3	45000	1000	23000	-44000	
4	30000	500	15250	-29500	
5	25000	600	12800	-24400	
6	42000	900	21450	-41100	
7	38000	800	19400	-37200	
8	32000	700	16350	-31300	
9	29000	650	14825	-28350	
10	47000	950	23975	-46050	
11	41000	850	20925	-40150	
12	36000	750	18375	-35250	
13	44000	950	22475	-43050	
14	31000	600	15800	-30400	
15	27000	700	13850	-26300	
16	48000	1100	24550	-46900	
17	43000	850	21925	-42150	
18	33000	800	16900	-32200	
19	29000	650	14825	-28350	
20	50000	1200	25600	-48800	
